# Gnathostomiasis Acquired by British Tourists in Botswana

**DOI:** 10.3201/1504.081646

**Published:** 2009-04

**Authors:** Joanna S. Herman, Emma C. Wall, Christoffer van Tulleken, Peter Godfrey-Faussett, Robin L. Bailey, Peter L. Chiodini

**Affiliations:** Hospital for Tropical Diseases, London, UK (J.S. Herman, E.C. Wall, C. van Tulleken, P. Godfrey-Faussett, R.L. Bailey, P.L. Chiodini); London School of Hygiene and Tropical Medicine, London (P. Godfrey-Faussett, R.L. Bailey, P.L. Chiodini)

**Keywords:** Gnathostoma sp., gnathostomiasis, eosinophilia, Botswana, United Kingdom, tourists, bream, albendazole, ivermectin, dispatch

## Abstract

Infection with *Gnathostoma spinigerum* has been generally confined to Southeast Asia and Central and South America. However, gnathostomiasis was recently found in British tourists who had visited Botswana. Consequently, travel to Africa should now be considered a risk factor for gnathostomiasis.

In recent years, gnathostomiasis has increasingly been found in persons in countries where *Gnathostoma spinigerum* has not been endemic. However, *Gnathostoma* spp. should now be considered emerging imported pathogens. Apart from 2 previous reports of gnathostomiasis in Zambia and Tanzania (*1*), Africa has been considered free of this disease. Most persons seen in the West with gnathostomiasis have acquired the infection in Southeast Asia, particularly in Thailand and Japan (*2*–*4*), or in Central or South America, especially Mexico (*5*,*6*), where the main risk factor is consumption of raw or undercooked fish. Because few clinicians outside gnathostomiasis-endemic regions are familiar with the disease, diagnosis is often missed or delayed.

Our report describes a man with confirmed gnathostomiasis and another who probably had the disease; both had been on a fishing trip between Shakawe and Maun in northwest Botswana. A recent health alert from Johannesburg, South Africa, describes 2 clusters of infection with *Gnathostoma* sp. also acquired in the same region in Botswana as that visited by our 2 patients (*7*).

## The Cases

The first person evaluated for possible gnathostomiasis was a British Caucasian man, 41 years of age, who came to the Hospital for Tropical Diseases (HTD) in London on September 30, 2008, 3 weeks after his return from the Okavango Delta in Botswana. He had been camping, walking barefoot, and swimming in and drinking river water. On several occasions, he had also eaten raw bream. About 2 weeks later, he reported intermittent abdominal discomfort, which was localized, but each morning, the pain moved to a different site and was accompanied by a palpable swelling over his spleen that resolved after 12 days. Pruritus then developed under his left arm and, within 24 hours, a painful subcutaneous lump developed on his anterior chest wall.

When he arrived at HTD, he was systemically well but had a raised nontender erythematous lesion below his left axilla. His eosinophil count was slightly high, 0.69 × 10^9^ cells/L (reference count <0.4 × 10^9^ cells/L).

A presumptive diagnosis of migratory helminthic infection was made (gnathostomiasis or larval cestode infection), and he was treated with ivermectin, 200 μg/kg as a single dose, and albendazole, 400 mg 2×/d for 21 days. A week later, the lesion had migrated to his neck, but within 14 days, the lesion and eosinophilia had resolved. His initial serologic test result was negative for *Gnathostoma* spp. A subsequent sample was also negative, which may indicate that the antibody response had not developed sufficiently or that results were outside the sensitivity range.

A second British Caucasian man came to HTD on October 11, 2008, eleven days after the patient previously described, and 5 weeks after the new patient’s return from the same trip in Botswana, where he had consumed the same foods and participated in the same activities. An erythematous, edematous, and pruritic lump (2 cm) developed above his left groin, lasted 4 days, and then subsided. As the lump decreased in size, he noticed a histamine-type track (left by a larva moving through tissue) toward his ribcage. One week later, the track mark had moved further up his chest. After another week, he reported a swollen, warm, and itchy right knee, which resolved within 24 hours, but 7 days later, similar symptoms developed in his right ankle.

**Figure 1:**
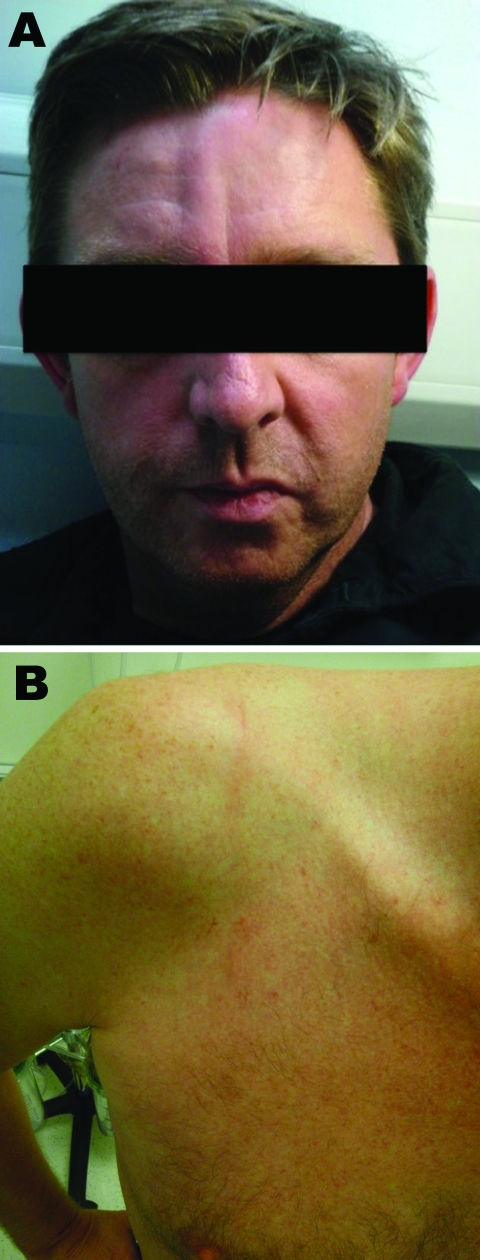
Cutaneous larva migrans on the forehead (A) and shoulder (B) of a male British tourist who had visited Botswana.

He visited HTD again with a serpiginous, raised lesion on his back and surrounding erythema and eosinophilia (0.9 × 10^9^ cells/L). He was treated empirically with albendazole, 400 mg 2×/d for 21 days, and praziquantel, 20 mg/kg as a single dose, for presumptive diagnosis of helminthic infection. Over the next 6 days, the serpiginous lesion migrated over his shoulder and neck, disappeared for 24 hours, then reappeared between his eyebrows, moved to his forehead and face, and then was felt inside his nose (Figure 1). On day 6, a spot developed below his left nostril, from which he expressed a larva. He brought it to HTD, where it was identified as *Gnathostoma spinigerum*.

## Conclusions

Our patients received a diagnosis of typical cutaneous gnathostomiasis, but their lack of travel to a region in which the disease was known to be endemic was perplexing. The only previous reports of this infection from Africa were in 3 persons who ate raw catfish from the Rufiji River in Tanzania (1994) and in 2 travelers who ate raw bream from the Zambezi River in Zambia (1998) (*1*). Before these reports, all reported cases of gnathostomiasis had been acquired in Southeast Asia and Central and South America.

Gnathostomiasis is a nematode infection caused by the late third-stage larva (L3) of the helminth *Gnathostoma* spp. A foodborne zoonosis, it is endemic where people eat raw or undercooked fish that harbor the infectious L3. At least 4 species are known to cause human disease, the most common being *G. spinigerum*. Adult nematodes live in the stomach of definitive fish-eating hosts (commonly cats and dogs).When feces containing eggs are deposited in water, free-swimming first- and then second-stage larvae develop (*2*,*8*). Ingested by the first intermediate host (a copepod), they develop into early L3 forms. Second intermediate hosts (freshwater fish such as bream, catfish, snake-headed fish, sleeper perch, Nile tilapia, butterfish, or eel; frogs; snakes; chickens; snails; or pigs) ingest the copepods, liberating the larvae, which encyst in muscle and mature into L3 forms. When infected fish are eaten by a definitive host, the larvae mature into adults in ≈6 months (see life cycle of *G. spinigerum*, adapted from an illustration by Sylvia Paz Diaz Camacho, available from www.dpd.cdc.gov/dpdx/HTML/gnathostomiasis.htm). Humans are accidental hosts in which the parasite fails to reach sexual maturity.

Two alternative routes of human infection have been suggested: ingestion of water that contains infected copepods or direct skin penetration of food handlers through L3-infected meat (*2*,*8*). Symptoms in humans occur as the larva migrates through tissues, causing cutaneous and/or visceral larva migrans, which may begin within 24–48 hours after ingestion of infected meat. Initial nonspecific symptoms include fever, malaise, nausea, vomiting, diarrhea, and epigastric pain lasting 2–3 weeks and usually accompanied by a marked eosinophilia. Within 1 month, the cutaneous form may develop, with characteristic nonpitting edematous migratory swellings that may be painful, pruritic, or erythematous and may last 1–2 weeks. The swellings are typically due to 1 larva, occasionally due to 2 or more (*8*). Spontaneous larval extrusion, such as occurred in 1 of our patients, has been recorded.

Visceral gnathostomiasis occurs when the larvae migrate through the internal organs such as the lungs, gut, genitourinary tract, eye, ear, and central nervous system. This form causes more illness and deaths, with mortality rates reported at 8%–25%, than the cutaneous form (*9*,*10*). Pathogenicity is thought to result from direct mechanical injury by the larvae and by their release of secretions and excretions, which contain various compounds that cause tissue damage (*9*). The result is characteristic hemorrhagic tracts that may be seen at autopsy. Untreated, infected persons may have intermittent symptoms until the larvae die, after ≈12 years.

**Figure 2:**
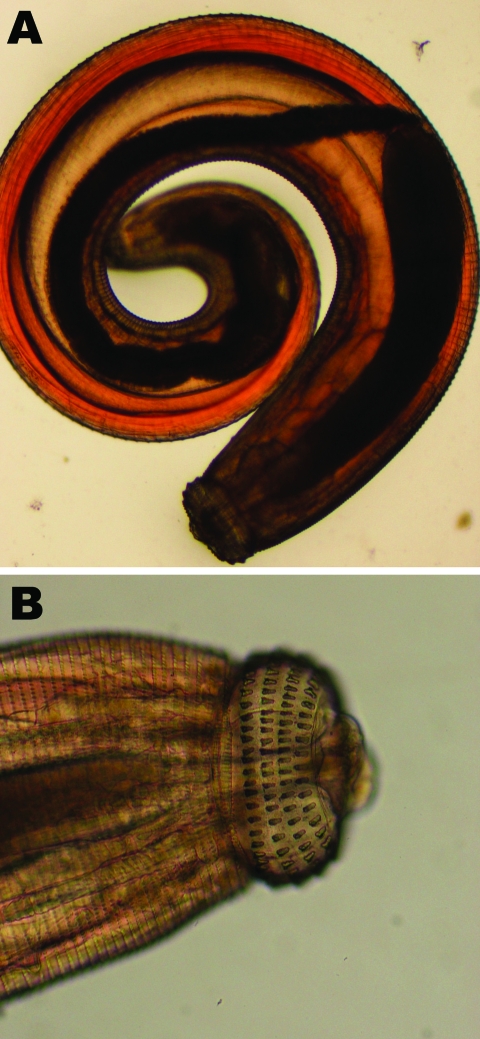
Third-stage larva of *Gnathostoma spinigerum*, which was expressed from the face of a male British tourist who had visited Botswana. Photograph shows entire larva (A) and larva head with hooks (B).

Diagnosis is suggested by eosinophilia, migratory lesions, and a history of geographic and food exposure. An immunoblot detecting 24-kDa–L3-specific antigen is considered diagnostic of *Gnathostoma* sp. and is the most widely used serologic test because of its high sensitivity and specificity (*11*; P. Dekumyoy, pers. comm). Some laboratories use an ELISA for immunoglobulin G subclasses as a screening test (*12*). In our first patient, the larva was identified based on its morphologic features, including the number and size of cephalic hooks and the character and extent of spines on its body (Figure 2) (*2*,*13*).

For many years, treatment with various drugs was unsuccessful, and surgical excision of the larvae was the only effective management. Studies in the 1990s confirmed the efficacy of a 21-day course of albendazole, 400 mg 2×/d (*14*,*15*), and ivermectin, 0.2 mg/kg immediately or for 2 consecutive days (*15*).

The area for risk of acquiring gnathostomiasis is expanding, and travelers and physicians need to be aware of this risk. Eradication of the organism is unlikely given its complicated life cycle, but local campaigns and appropriate pretravel advice can raise awareness and change people’s eating habits. Travelers must realize that culinary adventures on exotic holidays can result in acquiring unwanted parasites that may have devastating consequences.
